# The Evolution, Spread and Global Threat of H6Nx Avian Influenza Viruses

**DOI:** 10.3390/v12060673

**Published:** 2020-06-22

**Authors:** Holly Everest, Sarah C. Hill, Rebecca Daines, Joshua E. Sealy, Joe James, Rowena Hansen, Munir Iqbal

**Affiliations:** 1 The Pirbright Institute, Woking GU24 0NF, UK; holly.everest@pirbright.ac.uk (H.E.); rebecca.daines@pirbright.ac.uk (R.D.); joshua.sealy@pirbright.ac.uk (J.E.S.); 2 Nuffield Department of Medicine, University of Oxford, Oxford OX3 7BN, UK; 3 Department of Zoology, University of Oxford, Oxford OX1 3SZ UK; sarah.hill@zoo.ox.ac.uk; 4 Pathobiology and Population Sciences, Royal Veterinary College, Hertfordshire AL9 7TA, UK; 5 Department of Virology, Animal and Plant Health Agency, Addlestone KT15 3NB, UK; joe.james@apha.gov.uk (J.J.); rowena.hansen@apha.gov.uk (R.H.)

**Keywords:** H6Nx, avian influenza, zoonotic, pandemic, poultry, reassortment, virus evolution

## Abstract

Avian influenza viruses of the subtype H6Nx are being detected globally with increasing frequency. Some H6Nx lineages are becoming enzootic in Asian poultry and sporadic incursions into European poultry are occurring more frequently. H6Nx viruses that contain mammalian adaptation motifs pose a zoonotic threat and have caused human cases. Although currently understudied globally, H6Nx avian influenza viruses pose a substantial threat to both poultry and human health. In this review we examine the current state of knowledge of H6Nx viruses including their global distribution, tropism, transmission routes and human health risk.

## 1. Introduction

Influenza A viruses (IAV), including Avian Influenza Viruses (AIV), are members of the *Orthomyxoviridae* family and contain a segmented, ribonucleic acid (RNA) genome. Each genome consists of eight, negative sense, single stranded viral RNA (vRNA) segments enveloped in a host-derived lipid membrane with two surface glycoproteins: haemagglutinin (HA) and neuraminidase (NA). Differences in the antigenicity and phylogenetics of these surface proteins allow characterisation of AIV into subtypes H(x)N(y). Individual isolates are named according to the pattern: antigenic type (A, B, C, D), host of origin, geographical location, strain number and year of isolation [1]. This is then succeeded by H(x)N(y) (e.g., A/American Green-winged Teal/Ohio/18OS2656/2018 (H6N1)). Where the host of isolation is Human, the host of origin is omitted from the strain name (e.g., A/Taiwan/2/13 (H6N1)).

The influenza A virus genome encodes 10 core proteins and a variable number of accessory proteins. The genome is coated in viral nucleoprotein (NP) and adopts a twisted pan handle conformation with trimeric polymerase (PA, PB1 and PB2) attached, forming a ribonucleoprotein (RNP) complex. The RNP is encircled by the M1 matrix protein which is surrounded by a host-derived lipid bilayer envelope in which the HA and NA as well as the M2 matrix protein are embedded [2]. Additionally, the core non-structural protein (NS) encodes an mRNA transcript that is alternatively spliced to express two proteins involved in innate immune modulation and export of RNPs from the nucleus [3].

The primary natural host of IAVs are wild aquatic birds (mainly of the order Anseriformes) [4,5] with the exception of H17N10 and H18N11 which have exclusively been isolated from bats [6]. AIVs may also cause sporadic incursions in domestic poultry; evidenced by H7N1 outbreaks in Italy from 1990 to 2000, and additionally in humans and other mammalian species [7,8,9].

AIVs can be classified into two groups as a result of clinical disease or molecular signature within their HA segment; low-pathogenic avian influenza virus (LPAIV) and high-pathogenic avian influenza virus (HPAIV) [10]. Specifically, a virus is considered a HPAIV if it has an intravenous pathogenicity index (IVPI) in six-week old chickens of greater than 1.2, or causes at least 75% mortality in four to eight-week old chickens when birds are infected intravenously. The virus is also considered a HPAIV if there is a polybasic cleavage site in the HA segment; endogenous furin-like proteases activate the HA at polybasic cleavage sites to facilitate a systemic, and often fatal, infection. Only subtypes H5 and H7 have displayed this phenotype in natural isolates. Absence of a polybasic cleavage site within the HA classifies the virus as being LPAIV [11,12]. However, the presence of a di- or tri-basic cleavage site in the HA gene can also lead to enhanced pathogenicity [13]. LPAIV typically causes milder clinical disease in poultry, often associated with a fall in production measures (commonly a drop in egg production with abnormal eggs), with high morbidity (>50%) and low mortality (<5%) [14,15]. However, in some cases of LPAIV infection mortality can increase, especially in instances of concurrent or secondary infection with other diseases [14,16].

Globally H6Nx viruses are becoming an increasingly persistent burden for the poultry industry [17], with frequent introductions incurring large scale disease. H6Nx viruses also boast the most extensive host species range compared to other subtypes [18], and recent evidence, explored in this review, suggests that H6Nx viruses harbour several molecular signatures indicative of mammalian adaptation therefore posing a potential risk to human health. This review aims to summarise the global distribution, evolution, host tropism infectivity and transmissibility and human health risk posed by H6Nx AIVs.

## 2. History and Emergence of H6Nx Viruses

### 2.1. History of H6Nx Isolation and Detection

The H6 subtype has been isolated from wild aquatic, domestic aquatic and terrestrial avian species throughout the world. Whilst the first detection of H6 subtype AIVs is widely regarded in literature as being from a turkey in Massachusetts, United States of America (USA) in 1965 (A/turkey/Massachusetts/3740/1965 (H6N2)), the oldest sequenced isolate is from Canada in 1963 (A/turkey/Canada/63 (H6N2)) [17,19,20]. Thereafter, LPAI H6Nx viruses were reported in Taiwan in 1972; Australia, USA and Canada around 1974, and Hong Kong in 1977 [17].

### 2.2. Species and Geographical Distribution of H6Nx Viruses

H6 AIVs affect both domestic poultry and wild birds. H6Nx infections have caused sustained poultry infections (ducks and chickens) and circulate in live bird markets (LBMs) in China. Surveillance indicates that H6 viruses have become enzootic in domestic ducks of southern China, mostly as H6N6 viruses. An 8-year surveillance study (1998–2006), that included more than 36,000 wild birds from Europe and the Americas, found that H6 was the most abundantly detected influenza virus subtype [18]. H6Nx viruses now boast the broadest host range compared to any other subtype [18].

**Figure 1:**
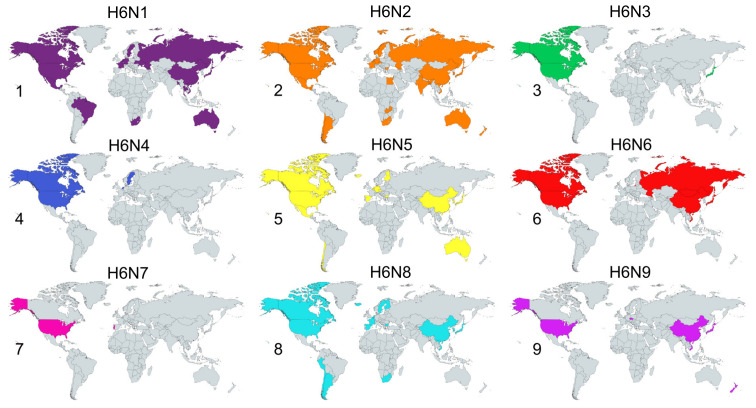
Countries that have sequenced H6Nx Avian Influenza Viruses (AIVs). All sequenced H6 viruses currently available on www.fludb.org were used to produce this figure. The maps correspond as follows: 1 = H6N1, 2 = H6N2, 3 = H6N3, 4 = H6N4, 5 = H6N5, 6 = H6N6, 7= H6N7, 8 = H6N8 and 9 = H6N9. Figure produced using www.mapchart.net.

H6Nx viruses are globally distributed (Figure 1) and have been isolated and sequenced with increasing frequency from domestic poultry, wild birds and several mammalian species including dogs and a single case in humans (Table 1) [21].

### 2.3. Global Surveillance of H6Nx Viruses

Global surveillance of LPAIVs, including H6Nx, is typically poor. LPAIV are never considered notifiable viruses in poultry and cause relatively few overt human infections, therefore, infections are not often investigated in detail. In many resource-limited regions, surveillance is not performed or performed only sporadically. LPAIV are only generally reported through research publications, genome announcements or WHO reports (in the case of confirmed human cases), and not via governmental surveillance. Therefore, it is highly likely that H6Nx viruses are present in far more countries than reported, including in low income regions not identified in Table 1.

## 3. Genomic Sequence Availability, Phylogenetics and Nomenclature

### 3.1. Availability of Sequence Data

**Figure 2:**
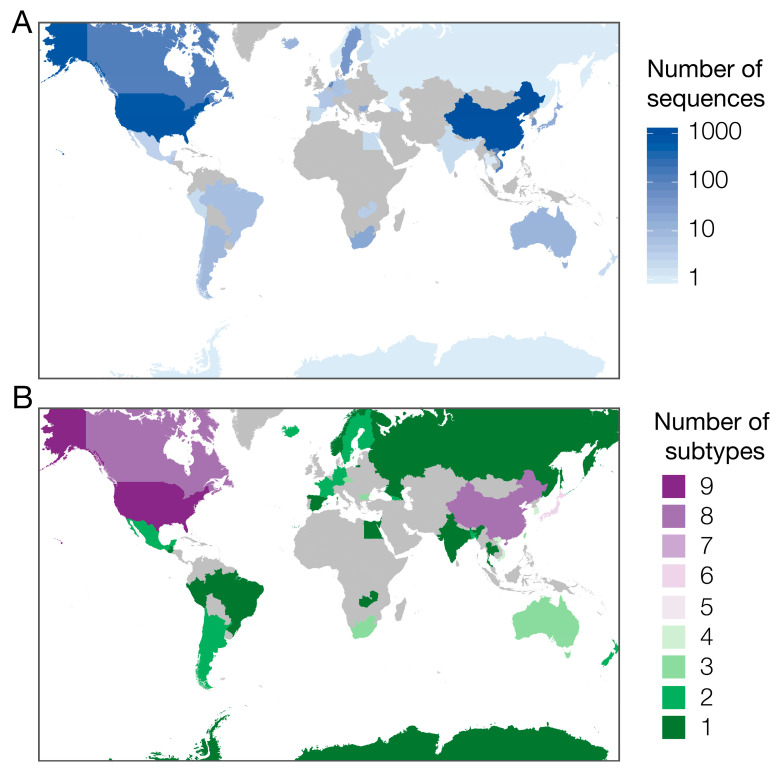
(**A**) Number of H6Nx sequences sampled in each country, (**B**) number of different H6Nx subtypes captured by available sequences. The countries represented in grey indicate no reported H6 HA sequence submissions.

As of 7 February 2020, 2501 full or majority partial (>70% of segment length) HA sequences were publicly available on FluDB (www.fludb.org). Most sequences are from Asia (55%, *n* = 1372), of which 76% (*n* = 1039) were sampled in China. Only 2% of sequences were sampled in South or Central America or Africa (*n* = 56) (Figure 2A), which may partly or wholly reflect lower surveillance and sequencing capacity in these regions. Increasing sequencing in these regions for H6 and other AIVs is important, particularly given projected regional intensification in the poultry industry [22].

**Figure 3:**
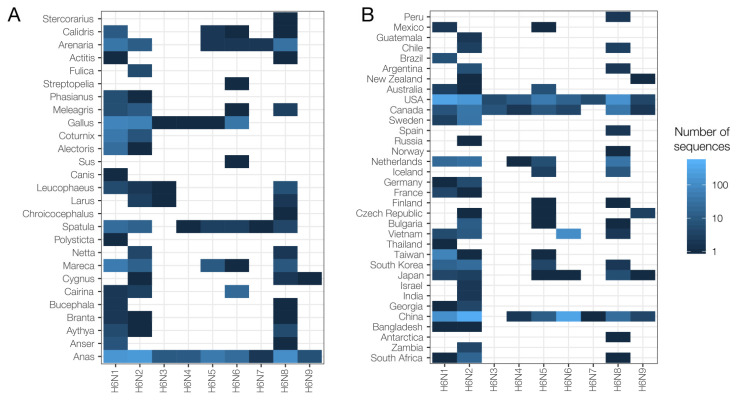
(**A**) H6Nx sequences by genera and subtype, for those subtypes where neuraminidase (NA) is known. (**B**) Sequences by country and subtype, for those subtypes where NA is known.

Subtype diversity of available sequenced strains is correspondingly higher in well-sampled regions (Figure 2B and Figure 3B), so absence of a sequenced subtype cannot be considered representative of true absence of that subtype in poorly-sampled regions. Nevertheless, the genetic sequence data available suggest that different NA subtypes of H6 AIV may be differentially prevalent across different hosts and countries (Figure 3). The apparent absence of whole genome sequences from H6N7, H6N8 and H6N9 in chickens (Figure 3A) and H6N3 in China is unusual, given the large numbers of sequences sampled from these groups. Whether this is caused by biased sampling, or by biological factors, should be investigated in greater detail.

### 3.2. Phylogenetics

**Figure 4:**
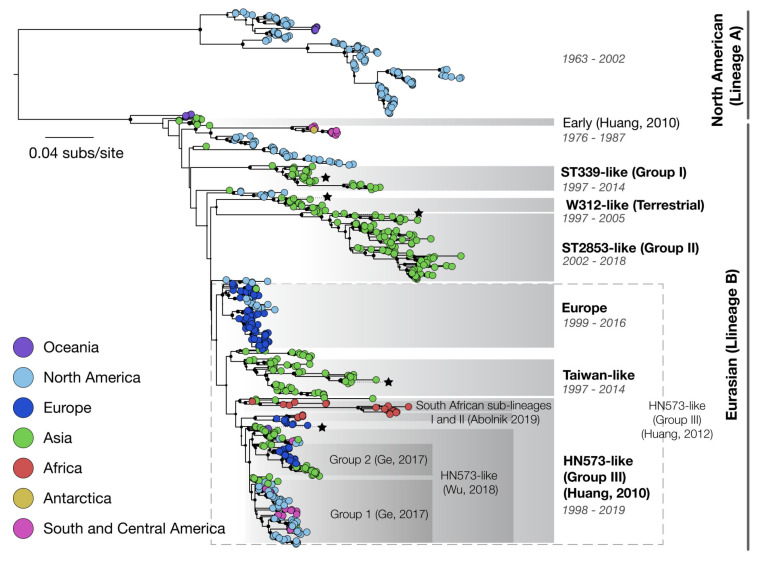
A representative phylogeny of currently available H6 HA sequences available on www.fludb.org All HA H6 sequences with >70% segment length (>1222 bp) were downloaded from FluDB (7/2/2020), with flagged sequences removed. The dataset was downsampled to select a maximum of five sequences from each year from each country, and commonly used lineage-defining sequences were included. MAAFT [27] was used to align the segments. A phylogeny was estimated using RAXML, using the best-fit GTR+G+I model as identified using modeltest-ng [28]. A total of 100 bootstraps replicate trees were constructed. Where specific clades in each named group was inconsistent between publications (e.g., HN573-like viruses), or lineages are identified in only one publication, specific publications are highlighted. Tips are coloured by location, and node size indicates bootstrap support for each node. Phylogenetic position of sequences used commonly to define lineages are given with stars. The most commonly used lineage nomenclature is highlighted in bold font.

Phylogenetic analyses of available sequence data have demonstrated that H6 HA sequence diversity is highly spatially structured, such that sequences sampled from the same country or continent tend to cluster together within the phylogenetic tree [23,24] (Figure 4). This is consistent with H6 viral movement in wild birds being concentrated within continental flyways, as has been previously reported, and is mixed and distributed globally [25]. Phylogenetic trees indicate that H6Nx reassorts extensively with AIVs of diverse NA and internal gene lineages (NA diversity indicated on Figure S1 [17,26].

### 3.3. Nomenclature

All AIVs have evolved into distinct lineages, typically characterised by geographical distribution. However, the rapid evolution and persistence of the H5 haemagglutinin (HA) derived from the A/goose/Guangdong/1996 H5N1 strain prompted the development of a standard clade nomenclature system, established by the WHO/OIE/FAO H5N1 Evolution Working Group. Phylogenetic analysis of H5 HA genes characterises sequences into clades based upon sequence homology and specific clade definition criteria. The nomenclature system is relatively fluid; as viruses within clades continue to evolve, new sub-lineages periodically emerge until the specific clade definition criteria are met, where they are then designated as separate clades [29]. Clades are defined through the sharing of a common (clade-defining) node in the phylogenetic tree; monophyletic grouping with a bootstrap value of ≥60 at the clade-defining node (after 1000 neighbour-joining bootstrap replicates); and average percentage pairwise nucleotide distances between and within clades of >1.5% and <1.5%, respectively [29].

There is no universally accepted or formal nomenclature system for H6 viruses, and those clades that have been widely recognised are loosely defined. The HA H6 segment can be initially phylogenetically divided at the root into Lineage A (sometimes termed “North American Lineage”) [21] that encompasses strains from North America and Oceania, and a Lineage B (“Eurasian Lineage”) that includes strains from all continental regions [30]. Several well-supported clades within Lineage B are widely recognised (bold text, Figure 4). Despite this, there is poor consistency between studies in exactly which phylogenetic nodes are basal to clades. Most notably, the term “HN573-like” viruses may refer to either a major clade [24], or instead to variously sized smaller sub-clades [23,31] (Figure 4). ST339-like viruses and ST2853-like viruses have both been interchangeably termed Group I and Group II (e.g., [17,23]). In addition, several well-supported clades have been identified and named within the literature, for example South African sub-lineages I and II (Figure 4) [32,33,34]. Sporadic and conflicting use of clade nomenclature between studies complicates scientific discussion of H6 evolution and diversity. To our knowledge, there has been only one attempt to propose a single global nomenclature, which analysed non-redundant H6 HA sequences between 2000 and 2014 [17]. Whilst the authors identified major clades Group I (mainly composed of H6N2 and H6N6 viruses from Asia) and Group II (which mostly consists of H6N1 and H6N2 viruses from Asia and around the world) and a single subdivision within one clade, the lack of a quantitative method for selecting named clades mean that the utility of this classification is limited.

The lack of nomenclature, or the essential requirement for one may be the result of a lack of available sequences for H6 viruses and a low rate of poultry outbreaks compared to other subtypes. Unlike H5 viruses, H6 viruses are not listed as a notifiable disease by the OIE, which may contribute to the lack of sequence availability and have a significantly lower incidence of human infections [12,35]. Additionally, despite persistence in wild bird populations, the predominant AIVs which are responsible for large-scale morbidity and mortality in poultry populations, H5, H7 and H9 subtypes, are prioritised in control strategies over H6 viruses. The increase in awareness, surveillance and reporting of H6 viruses may increase the availability of sequences which can be utilised to potentially establish a robust nomenclature system to monitor the evolution of these viruses. A single H6 nomenclature with clear classificatory guidelines should be proposed and adopted.

### 3.4. Molecular Epidemiology and Virus Evolution

Whilst the molecular epidemiology of H6Nx viruses has not been analysed in depth at a global scale, small scale regional analysis has been conducted.

Antigenic analyses of H6 influenza viruses isolated in south-eastern China between 1975–1981 and 1997–2000 has identified two antigenically distinct groups that continue to circulate in poultry [36]. Evolutionary genetic analysis of these viruses suggests that H6N1 viruses from south-eastern China have become adapted to terrestrial poultry [36,37]. It is suggested that these terrestrial 1999 and 2000 A/teal/Hong Kong/W312/97 (H6N1)-like viruses, along with co-circulating H9N2 viruses, could have been involved in the genesis of pathogenic H5N1 influenza viruses in 1997 that have caused substantial human infection [38].

## 4. H6Nx Viruses in Avian Species

### 4.1. Domestic Poultry Infections with H6Nx Viruses

**Figure 5:**
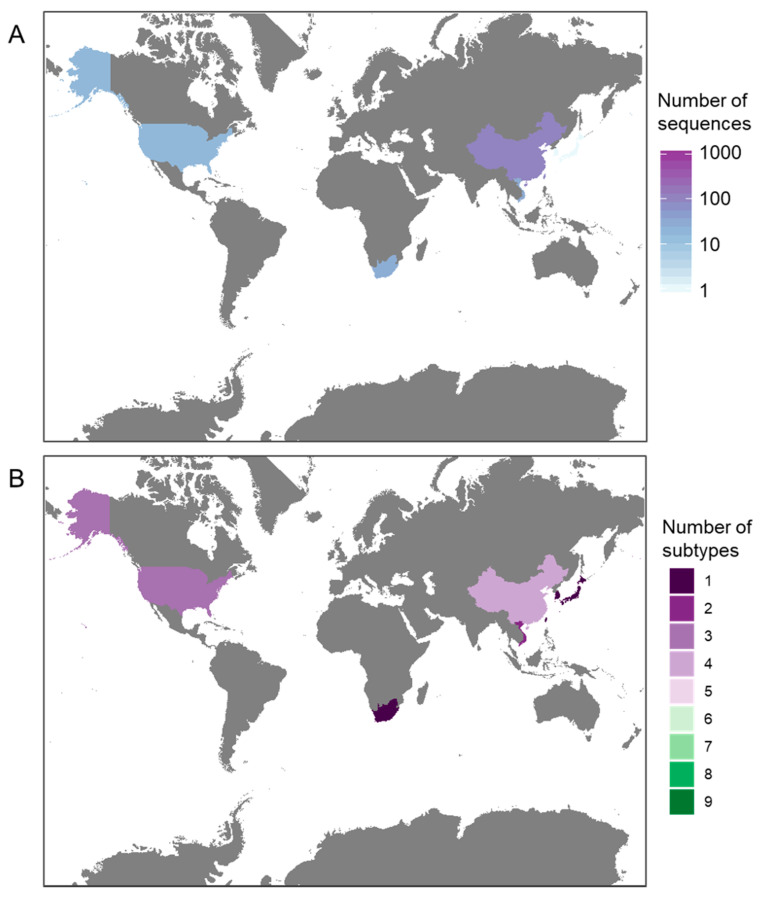
(**A**) A map indicating global H6Nx sequences reported in chickens. (**B**) A map showing the density of the number of different H6Nx subtypes in chickens. Most, if not all, chickens with reported AIV infections are domestic birds. Ducks, geese and turkeys have been excluded as there is no way to distinguish between whether they are of a domestic of wild nature.

H6 AIVs have been one of the predominant subtypes circulating since the 1970s in live bird markets (LBMs) in southern China and later South Korea, Vietnam, Cambodia, Thailand and Laos, and caused significant morbidity in domestic poultry. Domestic poultry can be defined as including chickens, turkeys, geese and ducks, that are raised for the production of meat or eggs. H6 continues to circulate in LBMs and is prevalent in several countries (Figure 5).

In the East Asian sub-region, H6N1, H6N2, H6N5, H6N6, H6N8 and H6N9 co-circulate in chicken and duck populations, undergoing frequent reassortment with other subtypes [23,39,40]. The migratory waterfowl reservoir is the most common source of H6 introduction into domestic poultry but is typically restricted in their ability to spread further [33]. H6 viruses typically cause asymptomatic infections in waterfowl but infection of chickens with H6 viruses are often associated with reduced egg yield, upper respiratory tract infection, morbidity and increased mortality [20]. Between February 2000 and February 2002, the California Animal Health and Food Safety Laboratory System diagnosed 26 cases of low-pathogenic H6N2 avian influenza from 12 commercial egg-laying farms (North American Lineage A, Figure 4) [41]. Clinical signs of H6N2 in layers range from nearly inapparent to relatively severe respiratory signs with egg yield reductions up to 30%. Weekly mortality increases between 1.6 and 5.1-fold with severe losses in broiler breeders reporting production losses up to 60% with further mortalities of up to 37% [33]. Some histological features present in chickens infected with H6N2 are fibrinous yolk peritonitis, salpingitis, oophoritis, nephritis and swollen kidneys [41].

### 4.2. H6Nx Infections in Avian Species in East and Southeast Asia

#### 4.2.1. Domestic Poultry

Southern China has been considered a hypothetical epicentre for facilitating the emergence of pandemic influenza viruses, with increasing diversity of the influenza ecology in the last two decades [42,43]. Surveillance in southern China from 2000 to 2005 indicated that H6 viruses were prevalent year-round in terrestrial poultry [44]. The earliest recorded isolates on FluDB from domestic poultry in Asia for ducks, is from Taiwan in 1972 (A/duck/Taiwan/0526/72 (H6N1)) [45] and for chickens, is from Hong Kong in 1977 (A/chicken/Hong Kong/17/1977 (H6N1)). However, due to the limited number of sequences available at the time, robust phylogeny and the identification of reassortants is extremely difficult to identify. Since 1972, infection with H6N1 LPAIV has also been prevalent in domestic poultry in Taiwan [46]. This is largely due to the preferred method of poultry trade in East and Southeast Asia. LBMs are a traditional component to poultry trading systems whereby live birds, which often includes chickens, ducks, quail and pigeons, are bought together in a single location from various different sources to ‘change hands’ or to be slaughtered. As a consequence, LBMs facilitate mixing of infected and non-infected birds which perpetuates the spread of AIV infection [20,41,46,47,48].

Domestic ducks are known to be particularly vulnerable to H6 AIV infections and more likely to be the major reservoirs for H6Nx viruses [17], when compared with chickens and geese [49]. A total of 16% (1325/8325) of samples collected from apparently healthy poultry in Southern China from 2000 to 2005 identified as H6 positive; 68.8% from aquatic poultry including domestic ducks and geese, 31.2% from minor poultry including partridges and quail, and a single isolate from chickens. H6N2 was the most prevalent subtype among all H6 viruses (79%), followed by H6N1, H6N5 and H6N6 [23]. A second study 2 years later found that the dominant H6N2 was replaced with H6N6 [24].

Domestic ducks act as intermediate hosts between the gene pool from migratory ducks and terrestrial poultry in the influenza virus ecosystem of this region [23,24]. Surveillance indicates that H6 viruses have become enzootic in the domestic ducks of southern China, mostly as H6N6 viruses [24].

Between 2011 and 2016, 11 surveys of AIV were performed in China that included almost the entire Chinese mainland. Epidemiological data from the study showed that the positive rate of H6 subtype AIVs in China increased each year from 2012 to 2016 [50]. From the perspective of host birds, the positive rates (from high to low) were geese (11.45%), ducks (3.81%), other poultry (0.57%), pigeons (0.26%) and chickens (0.11%), which is largely consistent with previous research. The positive rate of H6 AIVs in geese was significantly higher than it has been previously. This suggests that geese have begun to play an important role in the ecological distribution of H6 AIVs.

#### 4.2.2. Wild Aquatic Birds

The H6 viruses are among the most frequently encountered subtype in both wild and aquatic birds during surveillance programmes [23,24]. The majority of H6 duck isolates belonged to a distinct group with a single gene constellation, primarily comprised of H6N2 viruses. The emergence of this novel group was identified in Southern China, and cocirculates alongside viruses from the Eurasian natural gene pool [22]. The emergence and presence of this single distinct group is largely contradictory to other more recent surveillance studies in the region which have found a high diversity of isolated viruses across multiple subtypes, which is typically achieved by reassortment events. However, both opposing investigations identify the importance of both domestic ducks and wild aquatic avian species in the persistence of H6Nx infections in the region, acting as part of the natural reservoir along with wild aquatic birds but also as a major poultry type that harbours endemic virus lineages [23].

### 4.3. H6Nx Infections in Avian Species in Africa

#### 4.3.1. Domestic Poultry

South Africa’s H6N2 epizootic in chickens began in 2001 but was traced to a progenitor originating from a reassortment between viruses that infected commercial ostriches in the Western Cape Province in the mid to late 1990s, notably an H6N8 and an H9N2 virus [33]. The virus likely derived its M, NP, PB1 and PA genes from the H9N2 1995 ostrich virus A/Ostrich/South Africa/9508103 and the H6N8 virus, (A/Ostrich/South Africa/KK98/98) isolated in 1998 [32]. H6N2 is a controlled disease within South Africa and thus requires compulsory serological testing of registered compartmentalised commercial poultry farms and operations with significant contributions from non-commercial poultry flocks (e.g., backyard chickens) [33].

#### 4.3.2. Wild Aquatic Birds

Egypt is situated on important migratory flyways for wild birds between different continents [51]. Birds intended for sale in LBMs for human consumption were sampled and a novel reassortant strain of H6N2 was identified and isolated from a Eurasian Teal. This novel virus possesses a reassortant pattern originating from differing AIV gene pools. The complete genome sequence was obtained and designated as (H6N2) A/Eurasian teal/Egypt/P2-29/2017 (EG/P2-29) [4].

During AIV surveillance in Zambia (2008–2009) H6N2 was isolated from wild waterfowl. Phylogenetic analyses demonstrated that these viruses were of Eurasian lineage [52]. Some gene segments were closely related to those of AIVs isolated in Europe and Asia, indicative of reassortment and subsequent spread across continents. Analysis of the amino acid sequences of the internal genes revealed that several isolates contained genes of similarity predominantly observed in human influenza viruses [52].

### 4.4. H6Nx Infections in Avian Species in the UK and Europe

H6Nx viruses have also been obtained in both wild birds and commercial poultry flocks in Europe throughout the 2000s. Since 2001, H6N2 circulated in meat turkey flocks in Germany and H6N1 in infected meat turkeys was isolated in France [53,54].

In 2009 samples from birds at large commercial breeder farms in East and Southern England were tested for LPAI and H6N1 isolates were obtained. More recently, two separate outbreaks of H6N1 in commercial poultry have been detected in Northern Ireland and Scotland, respectively. H6N1 was isolated from a commercial farm in County Fermanagh in January 2020, resulting in substantial culling, following a previous reported case earlier in the month on a separate premise. In February 2020 a Scottish free-range laying flock were found to have been infected with LPAI H6N1, likely to have been contracted from wild birds. Further cases in Northern Ireland have occurred throughout February and March 2020 and similar outbreaks have been identified since in the republic of Ireland, Scotland and England.

Several cases of low pathogenic avian influenza H6 were identified in laying and turkey farms throughout the Netherlands between 2018 and 2019 [55]. Most cases were detected via monitoring (serologically positive) and a single case identified based on clinical symptoms. The circulating viruses appeared to be largely benign in poultry. The frequency of identification raises the question of whether specific strains of H6Nx may be becoming more adapted to poultry.

### 4.5. H6Nx Infections in Avian Species in the Americas

Inter-hemispheric movement of AIV genes from Eurasia to America have repeatedly been documented, with reverse movements less common [56]. This may be due to the high volume of migratory bird movements from Eurasia to America, the incompatibility of the Eurasian IAV gene pool with the introduced North American genes (or vice versa) or imbalanced viral surveillance monitoring. As a result, the North American AIV gene pool has permissibly been replaced by the Eurasian lineage within a decade of introduction [57].

#### 4.5.1. Domestic Poultry

H6N2 has caused sustained outbreaks among domestic poultry in the USA from 2000 to 2005 [41,58,59]. H6N2 influenza viruses have been isolated from chickens in California whose H6 genes do not fit into clearly defined North American lineages, which raised the question of whether Eurasian viruses are in the process of spreading to North America [56] and would subsequently cause reassortment. Between February 2000 and February 2002, the California Animal Health and Food Safety Laboratory System diagnosed 26 cases of low-pathogenic H6N2 avian influenza from 12 commercial egg-laying farms [41].

The most common gross and histologic lesions observed in California in chickens infected with H6N2 in 2000–2002, were fibrinous yolk peritonitis, salpingitis, oophoritis and nephritis. Oedema of the mesentery of the oviduct and pale, swollen kidneys were also observed. Mortality in infected flocks ranged from 0.25% to 3%, and egg production dropped 7% to 40%.

#### 4.5.2. Wild Aquatic Birds

AIV genome sequences (*n* = 109) from ducks in the Atlantic flyway were analysed to determine their genetic structure. The analyses included 25 AIVs from ducks from Newfoundland, Canada, from 2008–2011 and 84 available reference duck AIVs from the Atlantic flyway from 2006–2011 [60]. Of these 109 genome sequences, three H6 HA gene sequences were identified. The three H6 genes belonged to the lineage of Eurasian-avian origin, which is known to have become the predominant H6 lineage in North America in the last decade [56,60]. In contrast to the extensive animal influenza virus surveillance activities carried out in Southern China, East Asia, Northern Europe, Australia and North America, there is very limited data available from South America. Influenza A viruses from wild waterfowl in South America were rarely isolated and/or characterised. To explore the ecology of influenza A viruses in this region, a long-term surveillance program was established in 2006 for resident and migratory water birds in Argentina—a partnership between the National Institute of Agricultural Technology (INTA), the Argentinean National Animal Health Service (SENASA), the Wildlife Conservation Society (WCS), and the University of Maryland, College Park, in the United States. AIV surveillance is carried out for resident and migratory water birds, seabirds and shorebirds along the Argentine Atlantic coast and in freshwater wetlands formed by two of the major river systems in South America, the Paraná and Uruguay rivers [30]. Surveillance efforts are aimed at generating epidemiological data for an early warning of the introduction of AIV into commercial poultry.

H6 AIVs were isolated from hunter-killed ducks during the hunting seasons of 2007 and 2010. Specifically, rosy-billed pochards (*Netta peposaca*) in the Argentine wetlands were investigated and compared to prototypic H6 strains from North America and Eurasia. Of the five H6 isolates obtained, three were H6N2 subtypes and two H6N8 subtypes.

Analysis suggests the existence of an H6 South American lineage in which viruses do not appear to be mixing with and are evolving independently from influenza viruses in other latitudes [30]. One of the HA genes appears to have derived from a virus of North American lineage, indicating the presence of more than one H6 subtype cluster. Studies also suggest limited poultry adaptation and that additional molecular changes would be required for these viruses to replicate and transmit efficiently in chickens.

## 5. H6Nx Viruses in Mammalian Species and Zoonotic Infection

Most HPAIV or LPAIV infections in humans and other mammals have been associated with direct or indirect contact with infected live poultry or infected poultry products such as carcasses [61]. There has been no reliable evidence to suggest that the influenza viruses of avian origin are able to consistently or effectively transmit between mammals or humans following initial zoonotic spillover [61,62]. A prerequisite for AIVs to overcome the species barrier and become human-to-human transmissible is largely dependent on the HA and PB2 proteins. Human-to-human transmissible viruses should preferentially bind to α-2,6 sialic acid cell surface receptors, be able to withstand the harsh microenvironment of respiratory droplets and mammalian nasal tract, and be able to replicate efficiently in mammalian cells [63,64,65]. To date, naturally occurring H6 viruses have shown varied ability to bind to α-2,6 sialic acid receptors and a distinct lack of the PB2 E627K substitution, suggesting further mutation and/or reassortment is required for human-transmissible H6 viruses to occur; however, spillover events into mammals can and do occur [20,66].

Very little is known about the replicative capacity, immunogenicity, and correlates of protective immunity for low-pathogenicity H6 influenza viruses in mammals. H6 AIVs have been isolated from humans, pigs and a dog [67]. H6N2 along with H6N6 are viruses that are found to also replicate in mice without preadaptation, and some have acquired the ability to bind to human-like receptors [20].

### 5.1. Human Infections with H6Nx Viruses

On 21 June 2013, the first case of a human infection by an avian-origin H6N1 influenza A virus (A/Taiwan/2/2013, Taiwan2) was reported by the Taiwan Centres for Disease Control [21]. The Taiwan2 isolate was highly homologous to currently circulating chicken H6N1 viruses in Taiwan and had been generated as a result of interclade reassortment. The virus was also identified as LPAI due to the HA1/HA2 connecting peptide region (QIATR/GIF), lacking the multibasic amino acids.

Although the Taiwan2 infection was the first confirmed human case, H6-specific antibodies had been previously detected in personnel working in Chinese live-animal markets as well as U.S. veterinarians exposed to birds [68]. A total of 2124 serum samples, obtained from 1407 poultry workers from seven different areas of China between 2014 and 2016, were tested by haemagglutination inhibition and microneutralisation assays. Seroprevalence for H6Nx subtypes ranged from 0.4% to 2.5% [69]. Serological surveys conducted in both China and North America indicated that H6 influenza viruses might have previously infected swine and humans [68,70], and one-third of human volunteers inoculated with H6N1 or H6N2 viruses showed mild clinical symptoms with virus shedding [71].

The A/teal/Hong Kong/W312/97 (H6N1) and the human H5N1 and H9N2 influenza viruses possess similar genes encoding internal proteins, suggesting that H6N1 viruses could become novel human pathogens [36]. This A/teal/Hong Kong (HK)/W312/97 (H6N1) influenza virus is the first known isolate with seven A/HK/156/97 (H5N1)-like gene segments [36]. The A/teal/HK/W312/97 (H6N1) virus possesses not only these six internal protein genes but also the N1 neuraminidase (NA) gene of the human H5N1 influenza virus. This virus essentially represents the re-emergence of the H5N1 influenza viruses with a different hemagglutinin (HA) [72].

Though there is no evidence of inter-human transmission, the frequency at which humans appear to become infected raises the concern that avian H6 viruses may initiate human epidemics, if transmissibility between humans is gained.

### 5.2. H6Nx Studies in Laboratory Animals

Mice are a convenient and relatively cheap model in which to study of the mechanisms of influenza virus pathogenesis and human polymerase adaptation. H6Nx viruses are able to infect and replicate in mice without prior adaptation [20,73]. A specific example being reassortants between H6N2 and H6N6, two subtypes coexisting in LBMs in several provinces of Southern China. These viruses have affinity for human-like receptors and are also able to transmit between guinea pigs by direct contact [74].

Mouse infection studies conducted by Gillam Ross et al., with 14 different H6Nx viruses of North American and Eurasian lineage, showed that multiple viruses replicated in lungs of mice, causing varied degrees of morbidity and mortality [75]. However, the different H6Nx lineage viruses induced similar patterns of neutralising antibody responses. In general, neutralising antibodies from different H6 virus-infected mice were not capable of cross-reactivity between the different H6Nx lineages. However, a single H6 virus (A/teal/Hong Kong/312/97) was identified that induced protective immunity against viruses in the North American and Eurasian lineages [75]. This particular virus was identified as a potential precursor to the Hong Kong-origin H5N1 viruses isolated during the 1997 H5N1 outbreak in humans [72].

Ferrets are widely used animal models of human influenza, justified by the similar distribution of SA receptors in the upper respiratory tract to humans. They also exhibit similar clinical signs when infected with influenza [76,77,78,79]. Unlike for other zoonotic avian influenza subtypes, such as H5N1 [80], formal risk assessments based on ferret experimentation are not publicly available for H6Nx viruses. However, several ferret experiments for H6Nx infections have been conducted. In China, in recent years, frequent reassortments between influenza A(H1N1)pdm09 and other endemic SIVs have been observed. The internal genes of the influenza A(H1N1) pdm09 virus, which is prevalent in swine worldwide, increases the replication efficiency of H6N6 IAV in the lower respiratory tract of ferrets but not its transmissibility between ferrets. Experiments have shown that the virus has limited transmissibility through direct contact or through inhalation of infectious aerosolised droplets [81]. The aims of the study conducted by Sun et al. were to understand the impacts of mutations in HA of the endemic H6N6 virus on its receptor-binding properties and also to assess the transmission potential and potential risks posed by reassortants of H6N6 with the A(H1N1)pdm09 virus. Such reassortants have the potential to emerge naturally due to both viruses currently circulating in the swine population [82]. These findings suggest H6N6 swine IAV (SIV) currently poses a moderate risk to public health, but its evolution and spread should be closely monitored.

### 5.3. Canine Infection with H6Nx Viruses

A chimeric 1918 pandemic influenza virus expressing a contemporary H6 HA caused enhanced disease in mammals when compared to the chimeric virus alone, and inoculation of an avian H6N1 virus into dogs led to fever and detectable viruses in the lung [45].

Lin et al. [82] studied the prevalence of IAVs in dogs in Taiwan and identified A/canine/Taiwan/E01/2014 (E01/14). Molecular analysis indicates that this isolate is closely related to current avian H6N1 viruses circulating in Taiwan and harbours the E627K polymorphism in the PB2 protein. This polymorphism increases viral ability to replicate in mammalian species [82]. Phylogenetic analysis of the HA and NA gene segments confirmed that E01/14 belongs to the H6N1 lineage that has been circulating in chickens in Taiwan since 1997. Although the lineage of internal gene segments (PB2, PB1, PA, NP, M and NS) is composed mainly of H6N1 subtype viruses isolated in Taiwan, some H5N2 subtype isolates in the H6N1 lineage were observed [82].

### 5.4. H6Nx Virus Infections in Swine

The introduction of H6 AIVs from the natural reservoir into domestic poultry or mammals other than humans including pigs are typically through direct contact with the infected wild birds or indirectly through viral shedding into the environment via faeces and respiratory secretions [8]. Pigs play an important role in AIV infection and were proposed to be the “mixing-vessel” for the emergence of novel pandemic AIVs which were observed in 1957, 1968 and 2009, acting as an intermediate host for zoonotic transmission [8].

In 2010, a H6N6 virus emerged in southern China, and since then, has caused sporadic infections among swine, with the virus binding to both α2,6 (human-like) and α2,3 (avian-like) sialic acids [81].

## 6. Adaptation and Mutations in H6Nx Viruses

### 6.1. Mammalian Adaptation of H6Nx Viruses

Upon infecting a new host, the virus must successfully bind to, enter and replicate in the host cell as well as evade the innate immune response long enough for egress and subsequent transmission to a new host. Therefore, all these factors are essential in adaptation to a new host species. Several H6Nx viruses possess genetic substitutions that allow the virus to better bind to human receptors, replicate in human cells and better evade the human immune response (Table 2).

#### 6.1.1. Receptor Preference

The host adaptation of influenza virus is partly dependent on the sialic acid isoform bound by the viral HA. AIVs preferentially bind to sulphated or non-sulphated α2,3 isoform whereas human influenza viruses preferentially bind to the α2,6 isoform [65,86,87]. The receptor-binding specificity of influenza A viruses is also a major determinant for the host tropism of the virus, which enables interspecies transmission. H6Nx viruses have different propensities to bind α2,3 or α2,6 SA depending on the lineage.

Tzarum et al. [88] investigated the receptor specificity of the Taiwan2 human H6 HA using synthetic glycans and analysed the HA crystal structure. The Taiwan2 HA contains a unique combination of key residues in the HA receptor binding site when compared to avian origin H6 AIVs, which have never been observed in human or avian influenza viruses prior. Although amino acid residues 190V and 228S were thought to potentially promote human receptor binding, Tzarum et al. concluded that additional mutations may be required to change the binding specificity from α2,3 or α2,6 SA [88]. Further analysis of the human Taiwan2 H6N1 virus found that a single G225D mutation was able to alter the receptor preference from α2,3 to α2,6 SA [89].

#### 6.1.2. HA Mutations

The E190V and G228S mutations in the HA protein, as well as the frequent genetic reassortment of the H6 AIVs with zoonotic strains (e.g., H5N6), may increase affinity for the human-type receptor [61], which would pose an increasing threat to public health. Therefore, constant surveillance of AIVs in China, where multiple zoonotic strains are known to have originated, is needed to understand the evolution and dynamic reassortment of AIVs.

Using a panel of H6N1 viruses, Wang et al. demonstrated that the propensity of H6N1 viruses isolated from Southern China, to bind to human receptors, has increased between 1972 and 2013 [90,91]. By possessing mutations at the following positions, L186/V190/Q226/S228, there is an increase in human receptor binding affinity [66]. Mutagenesis work revealed that E190V and G228S substitutions are crucially important to acquire the human receptor-binding capacity, while the P186L substitution reduces the binding to α2,3 receptors [90].

Additionally, E190G produces a dual α2,3/α2,6 receptor binding phenotype with double E190D/G225D mutants having reduced binding to both α2,3 and α2,6 [92]. Approximately 34% of H6 viruses circulating in China have enhanced affinity to human-like receptors [20,89,93].

#### 6.1.3. NA Mutations

In a study conducted by Li et al. all N2, N3 isolates and 89.7% (*n* = 148) of the N6 isolates obtained from Southern China between 2014 and 2016, possessed a complete NA stalk, (470 amino acids) whereas 10.3% (*n* = 17) of the N6 isolates possessed an 11-amino acid deletion in the stalk region (positions 59–69). Interestingly, the H6N6 isolates with the N6-Δ11b (59–69) deletion were all clustered within the Asian H5N6 lineage. They were identified in ducks and geese, which suggested that the stalk deletion of the NA protein may not be the key factor determining the virus host range [24]. The phenotypic effect of these N6 NA deletions warrants further investigation.

#### 6.1.4. Internal Gene Mutations

Mouse infections with avian-origin H6N1 viruses found that polymorphisms PB2-E627K and PA-T97I in the viral RNA-dependent RNA polymerase (RDRP) complex increased the pathogenicity of H6N1 AIVs [73]. In vitro and in vivo analyses demonstrated that PB2-E627K and PA-T97I mutations significantly enhance RNA polymerase activity and viral replication thus contributing to enhanced replication in mammalian cells. E627K has also been shown to increase the replication of H5N1, H7N7, H7N9 and H9N2 AIVs in mammalian cells by interacting with the human isoform of ANP32 [92,94].

In a study conducted by Zou et al. all analysed isolates shared several molecular characteristics associated with AIV virulence, including E627 and D701 mutations in the PB2 gene [30], I38 in the PA gene; the nontruncated PA-X protein [95,96], and D92 in the NS1 gene. Additionally, S31 was found in M2 proteins in 191 H6 viruses, indicating no resistance to amantadine [67]. They also discovered that the expression of the PB1-F2 protein was blocked in 52.0% of the analysed H6 isolates, indicating the potentially variable virulence [97,98,99,100].

Two analysed isolates from Zambia, both H6N2, possessed the human-associated amino acid methionine at position 475 of the PB2 protein, which is described as being 100% conserved in the influenza viruses that caused the 1918, 1957 and 1968 human pandemics [101].

Serial passage of mice with a mallard origin H6N1 displayed mammalian adaptation, with increased pathogenesis caused by PB2-E627K and PA-T97I mutations in the viral RDRP complex [73].

### 6.2. Poultry Adaptation of H6Nx Viruses

Influenza viruses of terrestrial poultry origin differ from ancestral duck viruses by enhanced binding to different receptors. It is suggested that the adaptation to receptors in poultry can enhance the potential of an avian virus for avian-to-human transmission and pandemic spread [102]. The repertoire of glycan sugars in chickens differs from that of aquatic birds. Both α2,6- and α2,3-linked sialic acid receptors are present in the chicken nasal cavity, upper respiratory tract, and gut, whereas in ducks α2,3-linked sialic acid outweighs the proportion of α2,6 receptors [103,104,105,106]. During poultry adaptation the binding properties of the HA evolves [45], which may enhance the ability of the virus to infect cells in the human airway [79]. Conversely, these adaptations that induce fitness in terrestrial poultry may alter the HA–sialic acid interaction which could adversely affect the ability of that HA to support virus transmission in humans [107].

A study by Long et al. hypothesises that the acquisition of glycosylation on the HA head domain is a common evolutionary change in chicken-adapted avian influenza viruses. This in turn may decrease the strength of binding between virus HA and cell surface sialic acid receptors by steric hindrance [79,107].

The evolutionary dynamics of chicken-origin H6N2 viruses isolated in South Africa between 2002 and 2013 were investigated [34]. Sub-lineages I and II continued to co-circulate under vaccination pressure, but sub-lineage I, from which the inactivated vaccine was derived, displayed a markedly higher mutation rate and a three-fold increase in the emergence of potential antigenic sites on the globular head of HA compared to sub-lineage II. Immunological pressure culminated in a critical phenotypic change with several isolates losing the ability to hemagglutinate chicken erythrocytes. Co-assortment of the HA, NA and M genes in the respective sub-lineages contrasted reassortment of the other internal protein genes, and the vaccine seed strain itself was the probable donor of segments to sub-lineage II field strains [34].

Wang et al. [66] found that the chicken-H6N1-190E-228G HA mutant does not have as strong binding affinity as the duck-H6N1 HA does, indicating that other amino acid substitutions in the receptor-binding site could alter the binding affinity to avian-like receptors. They also found that recent biological characterisation of 256 H6 viruses from LBMs in China revealed that around 34% of H6N2 and H6N6 viruses could bind human-type receptors while maintaining an overall preference for avian receptors [66]. Yang et al. [108] performed a similar study to Tzarum et al. [88] using the same Taiwan2 HA and an avian H6 HA for comparison. Functional and structural data for the Taiwan2 HAs were very similar. The avian H6 HA was found to bind better and to a broader range of α2,3-SAs than the human H6 HA [108].

The HA of the 1999 and 2000 terrestrial viruses do not contain a multibasic cleavage site; but do contain a unique insertion of aspartic acid (D) in HA1 between positions 144 and 145 (H3 numbering). The NA of these terrestrial H6N1 viruses have a deletion of 19 amino acids which is a key feature of A/Hong Kong/156/97 (H5N1). Amino acid deletions within the NA stalk are a marker of adaptation from ducks to chickens for other subtypes, including H9N2.

Neuraminidase phylogeny showed that all N6 genes from Chinese poultry belonged to the Eurasian lineage and likely originated from H6N6 strains circulating in Eastern China during 2005 and 2006. Additionally, phylogenetic analysis identified that the PB2 gene of H6 AIVs were found in 52.5% of H5N6 AIVs isolates. Only 2% of the aquatic poultry tracheal swab samples were positive (16/911) with the other 98% of viruses isolated from cloacal or faecal swabs. This suggests that the H6 viruses isolated from this study primarily replicate in the gut [23] which is not usually considered typical of LPAI infections [109].

Antigenic and genetic characterisation of two novel H6N2 viruses isolated from apparently healthy domestic ducks in India between 2014 and 2015 revealed antigenic divergence between the two isolates; A/duck/India/11CL01/2014 (Kerala14) and A/duck/India/12CL22/2015 (Assam15). The sequence analyses indicated that both the viruses are of avian origin with α2,3 avian receptor specificity, low pathogenesis in poultry and sensitive to oseltamivir. However, Kerala14 (H6N2) had the V27I mutation marker indicating increased amantadine resistance in the M2 gene. Phylogenetic analysis revealed that both the viruses belonged to distinct clades and that both viruses are novel reassortants with genetically distinct gene constellations. The results suggest independent introductions of the two H6N2 viruses and that migratory wild birds in the Central Asian flyway may be the source of H6N2 viruses in ducks in India [34] and subsequently replicate how H6Nx have become prevalent globally.

## 7. Vaccination and Control

The circulation of influenza H6 subtype viruses in domestic birds highlights the need to develop an H6 vaccine to prevent potential influenza pandemics caused by the H6 viruses. The active circulation of the viruses and their ability to reassort with other AIVs, and subsequently their naturally distinct antigenicity could pose a challenge for vaccine development and selection in the event of a pandemic if novel H6 viruses were to arise [20].

A study by Chen et al. [110], based on the serum antibody cross-reactivity data obtained from 14 different H6 viruses from Eurasian and North American lineages, A/duck/HK/182/77, A/teal/HK/W312/97, and A/mallard/Alberta/89/85 were selected to produce live attenuated H6 candidate vaccines. Intranasal administration of a single dose of the three H6 candidate vaccine viruses (CVVs) induced neutralising antibodies in mice and ferrets and fully protected mice and ferrets from homologous wild-type (wt) virus challenge. Among the three H6 vaccine candidates, the A/teal/HK/W312/97 CVV provided the broadest cross-protection against challenge with three antigenically distinct H6 wt viruses. This data supports the rationale for further evaluating the A/teal/HK/W312/97 candidate vaccine in humans [110]. Data suggests that pandemic vaccines with receptor binding preference to both avian- and human-like receptors is desirable for efficient viral replication in eggs and for inducing protective immune responses in humans [111].

An inactivated whole virus vaccine used in South Africa, prepared from a 2002 sub-lineage I seed strain (Figure 4) was introduced to protect birds against clinical disease, but the continued use of this vaccine has previously been shown to accelerate the antigenic drift within sub-lineage I isolates [34]. H6N2 viruses in South African chickens are mutating and reassorting amongst themselves but have still remained as a genetically pure lineage (South African sub-lineages I and II (Abolnik et al. 2019), Figure 4) since their first emergence more than 18 years ago [33]. Greater efforts need to be made towards the continuous isolation and characterisation of field strains for use as Haemagglutinin Inhibition antigens, new vaccine seed strains and to monitor the zoonotic threat of H6N2 viruses [33].

## 8. Conclusions and Perspectives

In recent years, outbreaks caused by H6Nx infections have becoming increasingly frequently identified. Due to the vast geographical distribution of cases, across all nine NA subtypes, and its non-notifiable status, the true extent of H6Nx prevalence could massively exceed what is reported in literature. It causes sustained economic damage to the global poultry industry and is now endemic in several regions and multiple species.

As evidenced by successful isolation from a human infection, and significant seroconversion rates amongst poultry workers, H6Nx viruses pose a growing threat to both human and animal health. Although due to having LPAI classification, compared to HPAI viruses, there is a relatively low mortality rate with mild disease, there is a very high reassortment rate with proven adaptive changes. H6Nx infections are becoming more poultry adapted and have demonstrated ability to change receptor binding preference in several mammalian species. There is also the pandemic potential due to various viruses acting as progenitor and precursor strains to zoonotic viruses; e.g., H6N1 and the 1997 HPAI H5N1.

H6Nx viruses have a broad host range from which isolates have been successfully obtained—including from swine—which is of particular interest due to their susceptibility to both human and avian-origin influenza viruses, and their increased risk for mammalian adapted reassortant viruses and subsequent pandemic potential.

Both the prominence, and increasing frequency of, H6Nx infections highlights the warranted need for increased surveillance efforts globally and particularly throughout the UK and Europe, where multiple outbreaks of H6N1 have been particularly problematic in recent weeks. This surveillance should also be continued in endemic regions, especially in poultry workers and vaccinated poultry. Additionally, the need for a concise and globally recognised lineage and nomenclature system is highly relevant and would allow better understanding of the potential threat of new reassortant and emerging H6Nx viruses.

## Figures and Tables

**Table 1:** All H6 haemagglutinin (HA) sequences currently available on www.fludb.org with the corresponding years of detection and sampling source.

Country	Period	Subtype	Species	No. of Sequenced Strains	Total
**Antarctica**	2011	H6N8	Brown Skua, Chinstrap Penguin	2	2
**Argentina**	2007–2011	H6N2	Silver Teal, Comb Duck, Rosy-Billed Pochard, Yellow-Billed Pintail	7	9
	2007	H6N8	Rosy-Billed Pochard	2	
**Australia**	1979	H6N1	Pacific Duck, Gray Teal	3	8
	1979	H6N2	Eurasian Coot	1	
	1972–1980	H6N5	Pacific Black Duck, Shearwater,	4	
**Bangladesh**	2015	H6N1	Duck (unspecified)	1	2
	2016	H6N2	Mallard	1	
**Belgium**	2008–2010	H6N1	European Herring Gull, Turkey	3	21
	2011–2016	H6N2	Mute Swan, Eurasian Teal, Mallard	5	
	2009	H6N5	Mallard	1	
	2010–2016	H6N8	Mallard, Ruddy turnstone, Tufted Duck, Coot, Duck (unspecified)	12	
**Brazil**	2012	H6N1	White-Rumped Sandpiper	6	6
**Bulgaria**	2008–2010	H6N2	Mule Duck	12	14
	2010	H6N5	Mule Duck	1	
	2010	H6N8	Mule Duck	1	
**Canada**	1981–2010	H6N1	Mallard, Blue Winged teal, Green-Winged Teal	10	88
	1978–2005	H6N2	Mallard, Pintail Duc, Shoveler Duck, Turkey, Blue-Winged Teal, Common Coot	47	
	1985–1990	H6N3	Mallard, Pintail Duck	8	
	1979–1989	H6N4	Mallard, Pintail Duck, Blue-Winged Teal	3	
	1978–2003	H6N5	Mallard, Pintail Duck, Blue-Winged Teal	12	
	1982–2010	H6N6	Mallard, Pintail Duck, Blue-Winged Teal, Widgeon, American Black Duck	6	
	1978–1996	H6N8	Mallard, Pintail Duck, Blue-Winged Teal, Redhead, Turkey, Green-Winged Teal	40	
**Chile**	2016	H6N2	Franklin’s gull, Kelp Gull	3	6
	2016–2017	H6N8	Upland Goose, Mallard, Kelp Gull	3	
**China**	1997–2017	H6N1	Bean Goose, Chicken, Chukar, Duck (unspecified), guineafowl, Mallard, Partridge, Pheasant, Quail, Shoveler Duck, Common Teal, Turkey	109	937
	2000–2017	H6N2	Wild Bird (unspecified), Chicken, Chukar, Duck (unspecified), Environmental sample, Bean Goose, Goose (unspecified), Guineafowl, Mallard, Partridge, Pheasant, Pigeon, quail, Common Teal, Turkey	462	
	2003–2017	H6N5	Duck (Unspecified), Mallard, Shoveler Duck	11	
	2004–2016	H6N6	Chicken, Duck (unspecified), Environmental Sample, Goose (unspecified), Mallard, Muscovy Duck, Pigeon, Turtledove, Swine	321	
	2000–2016	H6N8	Wild Bird (unspecified), Duck (unspecified), Environmental sample, Goose (unspecified), Mallard, Common Teal, Wild Duck	32	
	2001–2007	H6N9	Duck (unspecified), wild Duck	2	
**Czech Republic**	2009	H6N2	Mallard	3	8
	2007	H6N5	Mallard	2	
	2009–2010	H6N9	Mallard	3	
**Finland**	2007	H6N5	Mallard	1	2
	2007	H6N8	Mallard	1	
**France**	1997–2010	H6N1	Turkey, Muscovy Duck	5	22
	2000–2005	H6N2	Pekin duck, Turkey, Mallard	15	
	2001	H6N8	Turkey	2	
**Georgia**	2015	H6N1	Mallard	1	4
	2010–2015	H6N2	Mallard, Common Coot, Domestic Duck	3	
**Germany**	1968–1999	H6N1	Turkey, Duck (unspecified)	2	9
	1999–2002	H6N2	Turkey	6	
	1998	H6N5	Turkey	1	
**Guatemala**	2013	H6N2	Blue-Winged Teal	2	2
**Hong Kong**	1976–2003	H6N1	Chicken, Chukar, Duck (unspecified), Guineafowl, Pheasant, Pigeon, Quail, Common Teal, Waterfowl	38	69
	1977–2002	H6N2	Duck (unspecified), Goose (unspecified), Guineafowl, Quail, Silky Chicken	19	
	1977–2001	H6N4	Chicken, Goose (unspecified), Pheasant, Quail	5	
	1976–1980	H6N5	Duck (unspecified)	2	
	1997	H6N7	Goose (unspecified)	1	
	1977	H6N8	Duck (unspecified)	1	
	1977–1997	H6N9	Duck (unspecified), Goose (unspecified)	3	
**Iceland**	2011	H6N5	Greylag Goose	3	12
	2011	H6N8	Greylag Goose, Pink-footed Goose	9	
**India**	2014–2015	H6N2	Duck (unspecified)	2	2
**Israel**	2001	H6N2	Wild Bird (unspecified)	2	2
**Japan**	2004–2017	H6N1	Chicken, Duck (unspecified)	12	44
	2000–2017	H6N2	Duck (unspecified)	15	
	1980	H6N3	Gull (unspecified)	1	
	2006–2008	H6N5	Duck (unspecified), Wild Bird (unspecified)	5	
	2014	H6N6	Wild Bird (unspecified)	1	
	1996–2017	H6N8	Mallard, Wild Bird (unspecified), Whooper Swan, Duck (unspecified)	8	
	2001–2008	H6N9	Duck (unspecified), Swan (unspecified)	2	
**Mexico**	2008	H6N1	Northern Shoveler, Green-Winged Teal	2	4
	2007	H6N2	Mallard	1	
	2009	H6N5	Green-Winged Teal	1	
**Mongolia**	2011	H6N6	Common Teal	1	1
**Netherlands**	1999–2012	H6N1	Chicken, Eurasian Teal, Eurasian Wigeon, Greater White-Fronted Goose, Greylag Goose, Mallard, Common Teal	28	128
	2000–2014	H6N2	Barnacle Goose, Bewick’s Swan, Gadwall Duck, Eurasian Wigeon, Greater White-Fronted Goose, White-Fronted Goose, Greylag Goose, Mallard, Common Teal	36	
	2011	H6N4	Mallard	2	
	1999–2015	H6N5	Greater white-fronted Goose, Mallard, Turkey	9	
	2004–2014	H6N8	Barnacle Goose, Bewick’s Swan, Black-Headed Gull, Greater White-Fronted Goose, Bean Goose, White-Fronted Goose, Mallard, Common Teal	53	
**New Zealand**	2005	H6N2	Mallard	1	2
	2005	H6N9	Mallard	1	
**Norway**	2005–2010	H6N2	Mallard, Teal (unspecified)	13	14
	2009	H6N8	Mew Gull	1	
**Peru**	2010	H6N8	Egret, Sandpiper	2	2
**Portugal**	2006	H6N7		1	1
**Russia**	2006–2009	H6N1	Duck (unspecified), Teal (unspecified), Herring Gull	6	9
	2006–2011	H6N2	Mallard, Gull (unspecified)	2	
	2009	H6N6	Teal (unspecified)	1	
**South Africa**	2009	H6N1	Ostrich	1	21
	2002–2019	H6N2	Chicken	18	
	1998–2007	H6N8	Ostrich	2	
**South Korea**	2003–2017	H6N1	Wild Bird (unspecified), Duck (unspecified), Spot-Billed Duck, White Pekin Duck, Bean Goose, Mallard, Shorebird (unspecified)	19	64
	2002–2017	H6N2	Wild Bird (unspecified), Duck (unspecified), Spot-Billed Duck, White Pekin Duck, White-Fronted Goose, Mallard	35	
	2005–2018	H6N5	Wild Bird (unspecified), Duck (unspecified), Bean Goose, Mallard	5	
	2005–2014	H6N8	Wild Bird (unspecified), Spot-Billed Duck, Mallard	5	
**Spain**	2007	H6N5	Mallard	1	3
	2006	H6N8	Duck (unspecified)	2	
**Sweden**	2002–2005	H6N1	Mallard	4	48
	2000–2009	H6N2	Guillemot, Mallard, Eurasian Wigeon	38	
	2014	H6N4	Mallard	1	
	2005	H6N8	Mallard	5	
**Taiwan**	1972–2014	H6N1	Chicken, Dog, Duck (unspecified), Partridge, Human	78	82
	2004	H6N2	Duck (unspecified)	1	
	1989–2004	H6N5	Chicken, Shorebird (unspecified)	3	
**Thailand**	2005	H6N1	Duck (unspecified)	1	1
**Ukraine**	2010	H6N1	White-Fronted Goose	1	1
**USA**	1976–2018	H6N1	American Black Duck, America Green-Winged Teal, American Wigeon, Wild Bird (unspecified), Environmental Sample, Blue-Winged Teal, Canada Goose, Common Goldeneye, Duck (unspecified), Long-Tailed Duck, Gadwall Duck, Eurasian Teal, Glaucous Gull, Cackling Goose, Greater White-Fronted Goose, Greater Scaup, Goose (unspecified), gull (unspecified), Hawk, Laughing Gull, Lesser Scaup, Mallard, Mottled Duck, Northern Pintail, Northern Shoveler Duck, Ring-Necked Duck, Ruddy Turnstone, Sanderling, Sandpiper, Shorebird (unspecified), Snow Goose, Steller’s Eider, Turkey, White-Fronted Goose	349	962
	1986–2015	H6N2	American Black Duck, America Green-Winged Teal, American Wigeon, Wild Bird (unspecified), Black Duck, Environmental Sample, Blue-Winged Teal, Chicken, Cinnamon Teal, Duck (unspecified), Gadwall Duck, Eurasian Teal, Greater White-Fronted Goose, Herring Gull, Goose (unspecified), Japanese Quail, Laughing Gull, Mallard, Mottled Duck, Northern Pintail, Northern Shoveler Duck, Pintail, Quail, Redhead, Ruddy Turnstone, Shorebird (unspecified), Turkey	244	
	1989–2009	H6N3	Chicken, Herring Gull, Laughing Gull, Mallard	7	
	1982–2016	H6N4	Environmental Sample, Gull (unspecified), Laughing Gull, Mallard, Northern Pintail, Ruddy Turnstone, Sanderling, Shorebird	32	
	1975–2015	H6N5	American Black Duck, America Green-Winged Teal, American Wigeon, Blue-Winged Teal, Chicken, Duck (unspecified), Environmental Sample, Green-Winged Teal, Mallard, Northern Pintail, Pintail, Red Knot, Ruddy Turnstone, Turkey	58	
	1980–2018	H6N6	American Wigeon, Blue-Winged Teal, Duck (unspecified), Green-Winged Teal, Mallard, Northern Pintail, Ruddy Turnstone, Sanderling, Turkey	31	
	2000–2018	H6N7	American Green-Winged Teal, Blue-Winged Teal, Mallard, Ruddy Turnstone	5	
	1977–2016	H6N8	American Black Duck, America Green-Winged Teal, American Wigeon, Wild Bird (unspecified), Black Duck, Blue-Winged Teal, Bufflehead, Canada Goose, Chicken, Duck (unspecified), Domestic Duck, Wood Duck, Environmental Sample, Eurasian Teal, Ross’s Goose, Herring Gull, Knot, Laughing Gull, Lesser Scaup, Mallard, Northern Pintail, Northern Shoveler, Pintail, Ring-Necked Duck, Ruddy Turnstone, Shorebird (unspecified), Shoveler, Turkey	198	
	1975–2014	H6N9	Duck (unspecified), Long-Tailed Duck, Green-Winged Teal, Mallard	4	
**Vietnam**	2013–2018	H6N1	Chicken, Duck (unspecified), Muscovy Duck	6	174
	2007–2014	H6N2	Duck (unspecified), Environmental Samples, Mallard, Muscovy Duck	40	
	2010–2018	H6N6	Chicken, Duck (unspecified), Environmental Samples, Mallard, Muscovy Duck	124	
	2013–2014	H6N8	Duck (unspecified), Mallard	2	
	2011	H6N9	Duck (unspecified)	1	
**Zambia**	2008	H6N2	Duck (unspecified)	4	4

**Table 2:** Mutations impacting the virulence/receptor preference of H6Nx AIVs.

Protein	Strain	Subtype	Host ^#^	Mutation *	Effect	Ref
**HA**	A/Taiwan/2/2013	H6N1	Human	G225D	Increasing its affinity for the human α2-6 linked sialic acid receptors	[83]
				S228G	Used in triad to overcome the requirement for a hydrophobic residue and preferentially bind to human-like receptors	[74]
				N137		
				V190D		
				P186L	Binding preference for the human receptor analog	[17]
				Q226L	Increased affinity for human-type receptor	[67]
				V135S	Increased affinity for human-type receptor	
				T136	Increased affinity for human-type receptor	
				V190D	Increased affinity for human-type receptor	[17]
	A/swine/Guangdong/K6/2010	H6N6	Swine	A222V	Increased human α2-6 linked sialic acid receptor preference	[81]
				S228G	Increased human α2-6 linked sialic acid receptor preference	
	A/Eurasian teal/Egypt/P2-29/2017	H6N2	Avian	T160A	Increased binding to human-type influenza receptor	[51]
	A/swine/Guangdong/K6/2010 [GDK6-MA]	H6N6	Mouse	L111F	Increasing its affinity for the human α2-6 linked sialic acid receptors	[84]
				H156N	Increased plaque size on MDCKs have enhanced early stage viral replication (H156N only) and concurrent occurrence alongside PA-I38M creates a significantly more virulent variant which compensates for lack of PB2-E627K	
				S263R		
	A/chicken/Guangdong/S1311/2010	H6N6	Avian	I55V	Increased affinity for human-type receptor	[85]
				S137N	Allows the binding to both human- and avian-like receptors	[67]
				E190		
				G228S		
				G225D	Increasing its affinity for the human α2-6 linked sialic acid receptors	
				Q226L	Increased affinity for human-type receptor	
	A/environment/Jiangxi/02.05 YGYXG001/2015	H6N6	Avian	S228G	Increased affinity for human-type receptor	[17]
**PB2**	A/Mallard/San Jiang/275/2007 (MA-P8M3)	H6N1	Mice	E627K	Enhanced RNA polymerase activity and viral replication which contributes to mammalian adaptation	[73]
	A/canine/Taiwan/E01/2014	H6N1	Dog	E627K	Enhanced RNA polymerase activity and viral replication which contributes to mammalian adaptation	[82]
	A/Eurasian teal/Egypt/P2-29/2017	H6N2	Avian	V291I	Host specificity shift towards human	[51]
	A/duck/Zambia/03/08	H6N2	Avian	475M	Increased affinity for human-type receptor	[52]
	A/swine/Guangdong/K6/2010 [GDK6-MA]	H6N6	Mouse	E627K	Enhanced RNA polymerase activity and viral replication which contributes to mammalian adaptation	[84]
**PA**	A/Mallard/San Jiang/275/2007 (MA-P8M3)	H6N1	Mice	T97I	Enhanced RNA polymerase activity and viral replication which contributes to mammalian adaptation	[73]
	A/swine/Guangdong/K6/2010 [GDK6-MA]	H6N6	Mouse	I38M	Raised polymerase activity in vitro	[84]
**M1**	A/Eurasian teal/Egypt/P2-29/2017	H6N2	Avian	N30D	When occurring concurrently, creates increased virulence (as seen in H5N1)	[51]
				T215A		
**M2**	A/chicken/Guangdong/S1312/2010	H6N2	Avian	S31N	Increased amantadine and rimantadine resistance	[20]
	A/chicken/Guangdong/S1311/2010	H6N6	Avian	S31N	Increased amantadine and rimantadine resistance	[20]
	A/duck/India/11CL01/2014	H6N2	Avian	V27I	Increased amantadine resistance	[34]
**NS1**	A/Eurasian teal/Egypt/P2-29/2017	H6N2	Avian	P42S	Increased virulence	[51]
				N205S		
	A/wild duck/Shantou/2853/2003	H6N2	Avian	D92	Increased virulence	[17]
**PB1-F2**	A/Eurasian teal/Egypt/P2-29/2017	H6N2	Avian	N66S	Altered virulence and antivirus response in mice	[51]

* H3 numbering used; # Host the virus was isolated from in the relevant study: (e.g., (A/Mallard/San Jiang/275/2007 (MA-P8M3) H6N1), the mutation was identified when serially passaged in mice, not in the original avian isolate).

## Supplementary Materials

The following are available online at https://www.mdpi.com/1999-4915/12/6/673/s1, Figure S1: Phylogeny of H6 segments as Figure 4, but with tips coloured by NA type of that virus.
