# Supplementary material for: The Evolution, Spread and Global Threat of H6Nx Avian Influenza Viruses
Source: Viruses. 2020 Jun 22;12(6):673. doi: 10.3390/v12060673 (PMC7354632; doi:10.3390/v12060673)
Supplement: Supplementary file 1 [file viruses-12-00673-s001.pdf]

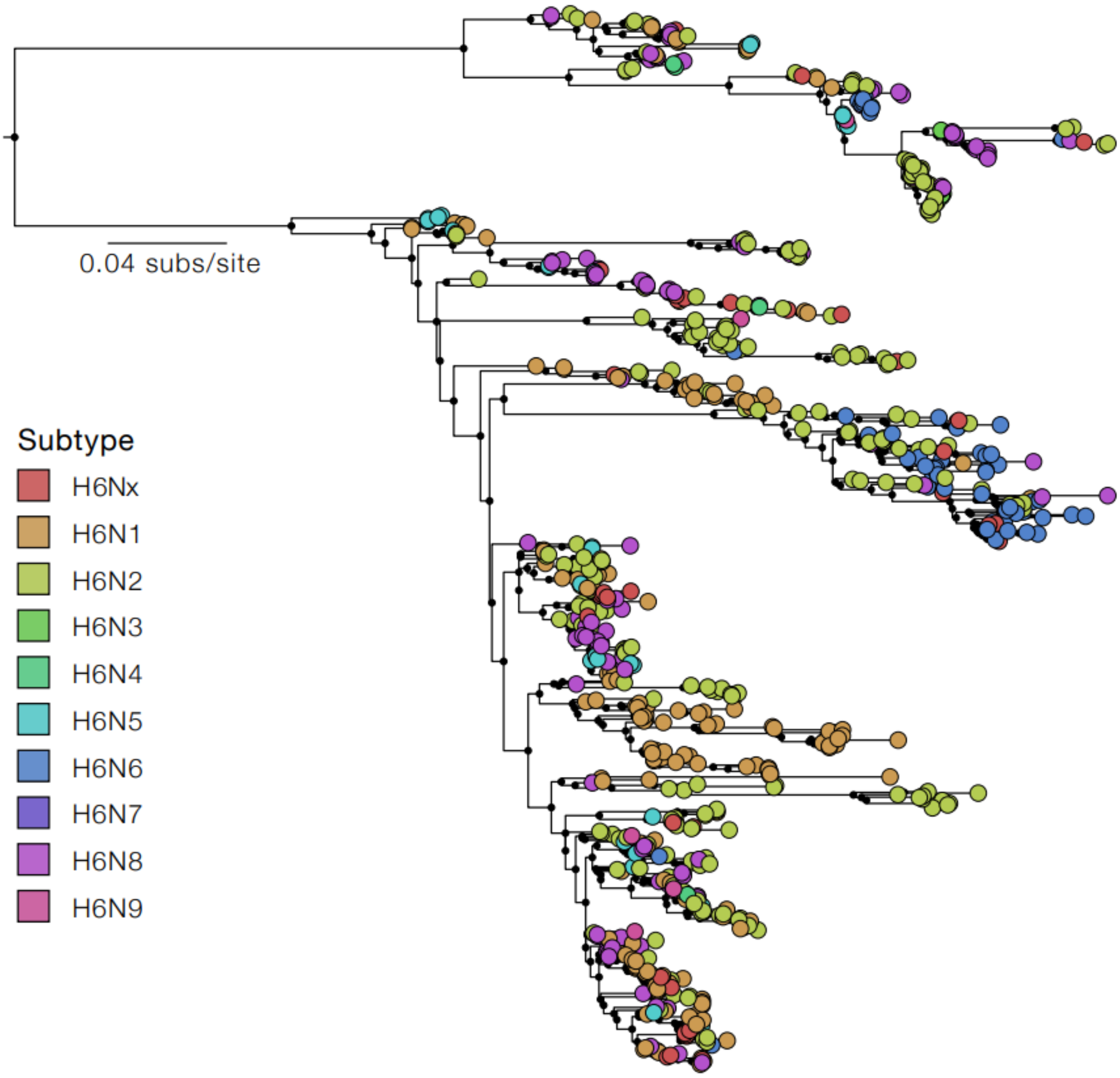

Supplementary Figure 1: Phylogeny of H6 segments as Figure 4, but with tips coloured by NA type of that virus.
